# Yield Enhancement of Valuable Lipid Compounds from Squid (*Doryteuthis gahi*) Waste by Ethanol/Acetone Extraction

**DOI:** 10.3390/foods12142649

**Published:** 2023-07-09

**Authors:** Santiago P. Aubourg, Alicia Rodríguez, Marcos Trigo, Isabel Medina

**Affiliations:** 1 Department of Food Technology, Marine Research Institute (CSIC), c/E. Cabello, 6, 36208 Vigo, Spain; mtrigo@iim.csic.es (M.T.); medina@iim.csic.es (I.M.); 2 Department of Food Science and Chemical Technology, Faculty of Chemical and Pharmaceutical Sciences, University of Chile, c/Santos Dumont, 964, Santiago 8380000, Chile; arodrigm@uchile.cl

**Keywords:** commercial waste, Patagonian squid, ethanol, acetone, waste/solvent, number of extractions, total lipids, phospholipids, tocopherols, ω3 fatty acids

## Abstract

The study focused on the extraction of valuable lipid compounds from squid (*Doryteuthis gahi*) waste by a low-toxicity solvent mixture (ethanol/acetone, 50:50, *v*/*v*). The effect of the waste weight/solvent volume (WW/SV, g·mL^−1^) ratio and the number of extractions (NoE) on the total lipid (TL), phospholipid (PL), and tocopherol yields and on the fatty acid (FA) profile (eicosapentaenoic and docosahexaenoic acid contents; polyunsaturated FAs/saturated FAs and ω3/ω6 ratios) was investigated. As a result, an increased NoE led to an increased (*p* < 0.05) TL yield but a decreased (*p* < 0.05) proportion of PLs in the lipid extract. Additionally, a lower (*p* < 0.05) polyunsaturated FA/saturated FA ratio was detected by increasing the NoE. Some differences (*p* < 0.05) could be outlined as a result of increasing the WW/SV ratio; however, a definite trend for this extraction condition could not be concluded for any of the lipid parameters measured. Yield results were compared to those obtained by the conventional chloroform/methanol procedure. In order to attain an increased yield, the NoE required would depend on the polarity degree of the lipid molecule concerned. All ethanol/acetone extracting conditions tested led to remarkable yields for lipid compounds (PLs, α-tocopherol, ω3 FAs) and FA ratios with healthy, nutritional, and preserving properties.

## 1. Introduction

Marine species are known as nutrient-rich and balanced foods because of their high content of valuable constituents [1,2]. Among them, the lipid fraction has acquired great attention as an important source of eicosapentaenoic (EPA) and docosahexaenoic (DHA) acids [3,4]. Both fatty acids (FAs) are especially concentrated in phospholipid (PL) classes and are reported to exert a positive influence on human health. This effect has been reported to be related to proper neural development, to the ability to see and learn, as well as to modulation of the eicosanoid synthesis and decreasing the risk of cardiovascular diseases (atherosclerosis, thrombosis, stroke), certain cancers, diabetes, depression, immune disorders, and others [5,6,7]. Marine species, especially oily fish, have been mentioned to include remarkable levels of tocopherol compounds [1,2]. Such compounds have also received great attention on the basis of their notable role as lipid-soluble chain-braking antioxidants [8,9].

Processing marine species generates great amounts of by-products. FAO states that annual seafood production contributes to over 180 million tonnes [10]; nonetheless, approximately a quarter of the total marine catch is discarded [11,12]. The valorization of discarded marine species and by-products of the seafood industry is among the top topics discussed in recent years because such substrates contain a similar constituent composition to commercial and edible tissues of marine species [13,14]. Recent studies have reported that many valuable components are present in marine by-products such as head, skin, viscera, and shell, and they could be incorporated into nutraceutical, functional food formulation, or pharmaceutical applications [15,16,17]. The development of new technologies and strategies for the recovery and purification of high-added-value compounds such as long-chain ω3 FAs, protein hydrolysates, collagen, chitin, or chitosan can promote better utilisation of discards and by-products.

Based on new challenges for the food industry, a wide range of eco-friendly methods are being used for lipid extraction from natural sources. Among them, ensilage [18], pH adjustment [19], wet pressing [20], enzymatic hydrolysis [21], supercritical fluid extraction [22], and urea concentration [23] have been employed satisfactorily. In order to replace petroleum-derived solvents, low-toxicity solvents such as ethanol, acetone, glycerol, or ethyl acetate have been tested for marine waste and marine substrates in general [24,25].

The Patagonian squid (*Doryteuthis gahi*) is a neritic species that is widely distributed along the Atlantic and Pacific coasts of South America [26]. After excision of the mantel tissue, all other body parts are considered by-products and are commonly assigned to meal production. However, previous research has shown a relevant presence of PLs, tocopherols, and ω3 FAs in Patagonian squid waste [27,28]. In such studies, different low-toxicity solvents and solvent mixtures were tested for their extraction. As a result, ethanol and acetone proved to be the most accurate solvents for PL and tocopherol extraction, respectively. However, previous research [27,28] pointed to a single waste weight/solvent volume (WW/SV) ratio and three-time extractions. The present study focused on the employment of an ethanol/acetone (50:50, *v*/*v*) mixture as an extracting system. To enhance the extraction of valuable lipid compounds and reduce the volume of the solvent employed, the effect of the number of extractions (NoE) and the effect of the WW/SV ratio were analysed.

## 2. Materials and Methods 

### 2.1. Initial Squid Waste and Lyophilization

Patagonian squid (*D. gahi*) waste, a mixture of heads, viscera, skin, tails, etc., was obtained from SERPESBA S. L. U. (Vigo, Spain). The initial squid samples were captured near the Argentinean coast in the south-west Atlantic Ocean, frozen on board (−40 °C), and transported for a 3–4-day journey to the Vigo (Spain) factory. Then, samples were thawed (overnight storage at 4 °C), the mantel was taken for commercialization purposes, and the resulting waste was pooled together and transported in refrigerated conditions (4 °C) to the laboratory, which is located in the same town.

Two × 1 kg of waste was lyophilized (−70 °C, 72 h, 0.05 mTorr) (Model FD8515-C60, Ilshin Biobase Europe, Ede, The Netherlands). The resulting lyophilized substrates were pooled together, minced, and used for moisture determination as well as lipid extraction by conventional and ethanol/acetone solvents according to the experimental procedure shown in Section 2.3.

Based on previous studies on the same squid species [27,28], the lyophilization step was found necessary to facilitate total lipid (TL) extraction by polar low-toxicity solvents and therefore increase the lipid yield. Additionally, moisture elimination has been described as necessary in previous research using relatively polar solvents such as acetone and ethanol for lipid extraction on other kinds of marine substrates [24,29].

Solvents and chemical reagents used were of reagent grade and purchased from Merck (Darmstadt, Germany); otherwise, the source is mentioned.

### 2.2. Moisture Determination and Conventional Lipid Extraction

The moisture content was determined in initial and lyophilized waste (1–2 g) according to the official AOAC method 950.46B [30]. The weight difference before and after heating (4 h at 105 °C) was determined. Results were calculated as g·kg^−1^ waste substrate.

The conventional lipid extraction of lyophilized waste was carried out according to the Bligh and Dyer [31] method. A single-phase solubilization of lipids was employed by using a chloroform/methanol (1/1) mixture. Lipid extracts were quantified according to the method proposed by Herbes and Allen [32]. Results were calculated as g·kg^−1^ dry waste.

### 2.3. Lipid Extraction with Ethanol/Acetone

The lipid extraction of lyophilized waste with ethanol/acetone (50:50, *v*/*v*) was carried out according to the extraction conditions described in Table 1. Two process variables were considered, i.e., WW/SV and NoE. Values taken into account were 0.20, 0.25, and 0.30 g·mL^−1^ for the WW/SV ratio and 1, 2, and 3 for the NoE. Each of the extraction conditions (from EC-1 to EC-9) was carried out in quadruplicate.

For each extraction condition, 3.0 g of lyophilized waste and a 10–15-mL volume of the ethanol/acetone system (Table 1) were mixed, stirred (1 min at 4 °C), centrifuged (3500× *g* for 10 min at 4 °C), and the supernatant was collected. This procedure was repeated one or two more times or not repeated according to the experimental design (Table 1). In each case, supernatants were pooled together. When necessary, a partial evaporation of the solvent mixture (rotary evaporator; 10 min at 30 °C) was carried out in all extracts. In all cases, extracts were brought up to a 15-mL volume and stored at −40 °C before analysis.

In all kinds of ethanol/acetone extracts, lipid quantification was carried out by the method proposed by Herbes and Allen [32]. Results were calculated as g·kg^−1^ dry waste.

### 2.4. Lipid Extract Analysis

The total PL content in the lipid extracts was measured spectrophotometrically at 710 nm (Beckman Coulter DU640 spectrophotometer, Brea, CA, USA), according to Raheja et al. [33]. In this method, a complex formation between PLs and ammonium molybdate is produced. For quantitative purposes, 1,2-dipalmitoyl-rac-glycero- 3-phosphocholine (Sigma-Aldrich, St. Louis, MO, USA) was employed as a standard. The results were calculated as g PLs·kg^−1^ lipids.

The profile of tocopherol compounds in squid waste was determined by the method proposed by Cabrini et al. [34]. For this purpose, the lipid fraction obtained from the lyophilized squid waste by each of the extracting systems (ethanol/acetone and conventional) was dried under nitrogen flux, dissolved in isopropanol, and analysed by HPLC (C18 5 μm, 4.6 × 250 mm column; XBridge, Waters, Milford, MA, USA). The column was fluxed with methanol for 2 min; then, a gradient from 0 to 50% isopropanol in 10 min was applied. A 1.5-mL·min^−1^ flow rate was employed, and detection was carried out at 292/328 nm in a HPLC Waters (mod. 2475, Milford, MA, USA) detector. The possible presence of α-, β-, γ-, and δ-tocopherol molecules was checked. For quantitative purposes, the content of each tocopherol present in the lipid extract was calculated with calibration curves prepared from the corresponding commercial tocopherol compound (Sigma–Aldrich) and calculated as mg·kg^−1^ lipids.

Acid-catalysed esterification and transesterification by employing acetyl chloride in methanol were applied to convert lipid extracts into fatty acid methyl esters (FAMEs). The resulting FAMEs were then subjected to gas–liquid chromatography (Perkin Elmer 8700 chromatograph, Madrid, Spain) analysis [35]. A fused silica capillary column SP-2330 (0.25 mm i.d. × 30 m, Supelco Inc., Bellefonte, PA, USA) was employed, programmed from 145 °C to 190 °C at 1.0 °C·min^−1^, from 190 °C to 210 °C at 5.0 °C·min^−1^, held for 13.5 min at 210 °C, and from 210 °C to 230 °C at 5.0 °C·min^−1^. Nitrogen at 10 psi as carrier gas and a flame ionisation detector (FID) at 250 °C were used. The qualitative analysis was carried out according to the FAME retention times and comparison to standard mixtures (Qualmix Fish, Larodan, Malmo, Sweden; FAME Mix, Supelco, Inc., Bellefonte, PA, USA). Peak areas were automatically integrated, and the quantitative analysis was carried out using C19:0 FA as the internal standard. The content of each FA was calculated as g·100 g^−1^ total FAs.

Results related to FA groups (saturated FAs, STFAs; monounsaturated FAs, MUFAs; polyunsaturated FAs, PUFAs; ω3 and ω6 FAs) and FA ratios (total PUFAs/total STFAs and total ω3 FAs/total ω6 FAs) were calculated on the basis of the results obtained for individual FAs.

### 2.5. Statistical Analysis

According to the experimental design described in Table 1, three different WW/SV ratios and three different NoEs were applied to the squid waste. Each of the extracting conditions (from EC-1 to EC-9) was carried out in quadruplicate (*n* = 4). Data obtained from the different lipid composition parameters (TL, PL, tocopherol, EPA, and DHA contents; PUFA/STFA and ω3/ω6 ratios) were subjected to one-way ANOVA (*p* < 0.05) to analyse the effect of the WW/SV ratio and the effect of the NoE on the yields and ratios obtained in the different ethanol/acetone extracting conditions (Statistica version 6.0, 2001; Statsoft Inc.). The comparison of means was carried out using a least-squares difference (LSD) procedure.

## 3. Results and Discussion

### 3.1. Moisture Values of Starting and Lyophilized Waste

The starting waste showed a moisture value of 83.24 ± 1.11 g·100 g^−1^. This value is similar to the one previously reported for the waste corresponding to the commercialization of the present cephalopod species [27,28]. The lyophilized powder resulting from the lyophilization step revealed a 1.95 ± 0.37 g·100 g^−1^ value of moisture.

### 3.2. Effect of the Extraction Conditions on the TL Yield

**Figure 1:**
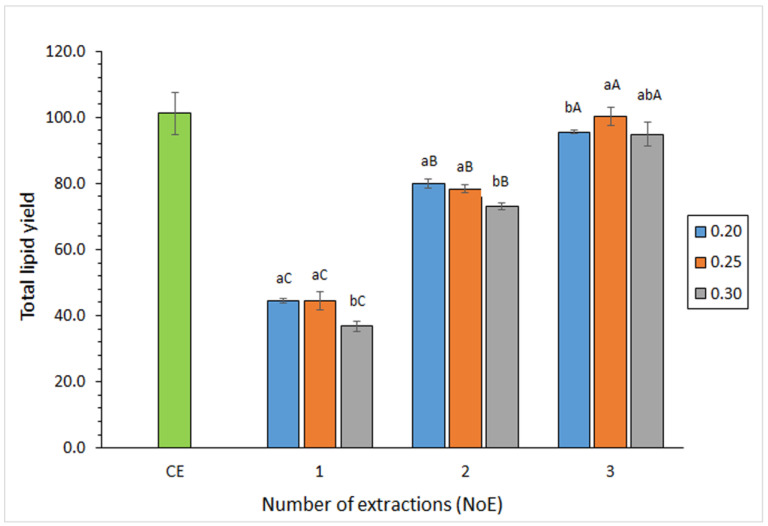
Effect of ethanol/acetone extraction conditions on total lipid (g·kg^−1^ dry waste) yield obtained from squid waste. Average values of four replicates (*n* = 4); standard deviations are indicated by bars. For each NoE (1, 2, or 3), different lowercase letters (a,b) denote significant (*p* < 0.05) differences as a result of the WW/SV ratio. For each WW/SV ratio (0.20, 0.25, or 0.30 g·mL^−1^), different capital letters (A,B,C) denote significant differences (*p* < 0.05) as a result of the NoE. Abbreviations: CE (conventional extraction), WW/SV (waste weight/solvent volume; g·mL^−1^), and NoE (number of extractions).

The NoE showed a great effect (*p* < 0.05) on the lipid yield obtained from the squid waste (Figure 1). Compared to the conventional procedure, yields corresponding to ethanol/acetone procedures were in the following range: 36–44%, 72–79%, and 94–99% for one-, two-, or three-time extractions, respectively. Regarding the WW/SV ratio (Figure 1), an increased value led to a higher lipid yield in the case of employing one- or two-time extractions. On the contrary, if a three-time extraction was applied, the highest average value was detected for a WW/SV ratio of 0.25 g·mL^−1^.

It is concluded that the current ethanol/acetone solvent mixture can lead to satisfactory lipid recoverability provided a three-time extraction and a WW/SV ratio of 0.25 g·mL^−1^ are carried out. Previous research investigated the lipid yield obtained from Patagonian squid (*D. gahi*) waste by employing different low-toxicity solvents (ethanol, acetone, ethyl acetate, and binary mixtures) [27]. In agreement with the present results, the highest lipid yield was obtained by using ethanol/acetone (50:50, *v*/*v*); however, yields were lower (ca. 71–81% compared to the conventional procedure) than in the current case. This lower lipid yield could be explained by the fact that we employed a different WW/SV ratio (i.e., 0.35 g·mL^−1^) than in the present study.

Previous studies focused on the lipid extraction from different marine waste substrates by employing different low-toxicity solvents and different eco-friendly procedures. Thus, the effect of previous heating on the lipid extraction by wet reduction of skipjack tuna (*Katsuwonus pelamis*) heads was studied [36]; as a result, lipid yields of 2.8 and 4.8% for precooked and non-precooked samples were obtained, respectively. An increased lipid yield was obtained by ethanol/hexane (2:1, *v*/*v*) extraction of hoki roe when compared to the conventional Folch procedure [37]; yields were found to be similar to those obtained by the less convenient two-step procedure (i.e., sequential ethanol and hexane extractions). A wet-rendering method at 121 °C was employed for lipid extraction from skipjack tuna (*K. pelamis*) eyeballs [20]; compared to the conventional procedure, the highest lipid recovery (ca. 64%) was obtained by employing a previous 20-min heating time. Different wet-pressing extraction procedures were carried out on squid (*Illex argentinus*) viscera [38]; optimised previous drying conditions (85 °C for 90 min) led to a ca. 76% yield when compared to the conventional extracting procedure.

Previous research has also addressed the employment of low-toxicity solvents for the lipid extraction of microalga substrates. However, the effect of the NoE and the effect of the microalga weight/solvent ratio were not studied. An increased presence of ethanol in an acetone/ethanol extracting system led to an increased lipid yield obtained from Antarctic krill (*Euphasia superba*) [24]. Notably, lipid yields obtained with 1/30 and 1/12 ratios were remarkably higher than those obtained with conventional procedures (i.e., Folch and Soxhlet methods). Lipid extraction with hexane/isopropanol (3:2, *v*/*v*) from microalga *Scenedesmus obliquus* was studied by applying subcritical conditions [39]; the optimised lipid yield was reached at 85 °C and 1.5 MPa conditions, leading to an 82.6% recovery when compared to the conventional procedure. Recently, Li et al. [25] carried out a comparative lipid extraction from microalga *Scenedesmus dimorphus* by employing several extracting systems (ethanol/hexane, 3:2; ethyl acetate/hexane, 1:1; methanol/hexane, 1:0.8; hexane; aq. 95% ethanol); according to the high PL presence in the microalga substrate, the highest lipid yields were obtained by applying 95% ethanol (ca. 90% yield when compared to the conventional extraction procedure).

### 3.3. Effect of the Extraction Conditions on the PL Yield

The effect of both extraction variables on the PL yield is depicted in Table 2. In all ethanol/acetone extracts, the PL content in the lipid extract was similar or even higher (ca. 98–106%) than in the one corresponding to the conventional procedure. An increase of the NoE led to a progressive decrease of the average PL value; however, differences were only found significant (*p* < 0.05) in the case of applying a WW/SV ratio of 0.25 g·mL^−1^.

Regarding the effect of the WW/SV ratio (Table 2), a definite trend could not be concluded. If a two-time extraction was applied, the highest (*p* < 0.05) PL values were detected for a WW/SV ratio of 0.30 g·mL^−1^; on the contrary, a 0.20 g·mL^−1^ value of the WW/SV ratio led to the highest average PL content when a three-time extraction was employed.

A decreased PL presence in the lipid extract by increasing the NoE value can be explained on the basis that the current ethanol/acetone mixture tested is more polar than the mixture employed in the conventional procedure. Thus, PLs would be more easily extracted than less polar lipid classes such as triacylglycerols (TAGs), waxes, cholesterol esters, etc. By repeating the extraction procedure (i.e., increasing the NoE), a more complete lipid extraction would be expected to occur according to the above-mentioned results on TL yields. This yield increase for TLs would lead to a relative decrease in the PL content in the TL extract.

PL classes have been described as being important constituents of cell membranes and therefore playing a decisive role in living bodies [6,40]. Based on their amphiphilic character, high bioavailability and absorption properties have been reported for PL compounds, leading to remarkable protective effects against ageing and chronic diseases [41,42]. A high PL presence in all kinds of lipid extracts was detected in the present study, so this kind of waste substrate can be considered a valuable marine PL source. Notably, a single-time extraction and a WW/SV ratio of 0.25 would be recommended in order to obtain the highest average PL presence in the ethanol/acetone lipid extract.

Previous research also focused on the PL extraction from squid (*D. ga*hi) waste by low-toxicity solvents [27]. In such a study, an increasing PL content was obtained according to the solvent sequence: acetone < ethyl acetate < ethanol. In agreement with the present study, this sequence was justified by an increasing polarity of solvents so that polar lipids such as PL classes would be better extracted. However, PL yields were found to be lower than in the current study and reached a ca. 85.8–90.3% range when compared to the conventional procedure. Such a difference with the present results can be explained on the basis that a higher WW/SV value (i.e., 0.35 g·mL^−1^) than in the present case was employed.

Previous studies focused on PL extraction from different marine substrates by employing eco-friendly and low-toxicity procedures. A higher PL yield (ca. 30%) was detected by applying a 1/6 ratio (acetone/ethanol) than in the case of using a higher presence of ethanol (1/9, 1/12, and 1/30 ratios, acetone/ethanol; ca. 21–22%) during PL extraction from krill (*E. superba*) [24]. By employing different eco-friendly extract procedures (95 °C for 30 min, <15 °C, and enzyme-assisted extraction), Głowacz-Rozynska et al. [43] found lower PL values (0.2–1.47 g·100 g^−1^ lipids) in salmon (*Salmo salar*) skins, heads, and backbones when compared to the present ones. With the aim of extracting high levels of polar-rich lipid fractions from microalga *Nannochloropsis* sp. biomass, a two-step extraction was carried out by Jiménez Callejón et al. [29]; thus, a hexane extraction of neutral lipids was followed by an ethanol extraction of the resulting pellet, leading to a highly-concentrated polar (ca. 87 g·100 g^−1^ lipids; i. e., PLs and glycolipids) lipid extract. The content of polar lipid compounds (i.e., phosphatidyl choline) showed an increase with previous heating time during the lipid extraction by a wet-rendering method of skipjack (*K. pelamis*) eyeballs [20]; thus, a ca. 4% presence in the lipid extract was reached after a 30-min heating time. An increased PL yield was obtained by the ethanol/hexane (2:1, *v*/*v*) extraction procedure on hoki roe by comparison to the conventional Folch method [37]; in the meantime, yields were found to be similar to those obtained by the less convenient two-step ethanol/hexane procedure.

### 3.4. Effect of the Extracting Conditions on the Tocopherol Content

As for PL compounds, α- and γ-tocopherol yields obtained with the current ethanol/acetone extracting mixture led to a high recovery when compared to the conventional procedure (ca. 93–113% and 96–190%, respectively) (Table 2). This higher content can be explained on the basis that non-polar lipid classes (i.e., TAGs and waxes) are not extracted entirely by the current low-toxicity solvents, leading to a relatively higher presence in the lipid extract of other lipid molecules such as α- and γ-tocopherol.

The extractability of both tocopherol molecules showed very similar tendencies. Thus, the NoE did not lead to a definite trend. For a WW/SV ratio of 0.25 g·mL^−1^, the highest α- and γ-tocopherol values (*p* < 0.05) in the lipid extract were obtained with a one-time extraction; on the contrary, for a WW/SV ratio of 0.30 g·mL^−1^, the lowest (*p* < 0.05) tocopherol contents were observed by applying a two-time extraction.

Regarding the effect of the WW/SV ratio (Table 2), the highest average tocopherol values were obtained for a 0.25 g·mL^−1^ value in the case of employing a one-time extraction. If two- or three-time extractions were applied, the highest average values were obtained for a WW/SV ratio of 0.20 g·mL^−1^.

According to previous related studies [28,44], α-tocopherol showed to be the most abundant tocopherol compound in the present squid waste. In the edible tissues of marine animals from natural diets, α-tocopherol has also been shown to be the most abundant [45]. On the contrary, the presence of β-, γ- and δ-tocopherol has been shown to be negligible in wild marine vertebrates and very low in invertebrates [45]. Current values for α- and γ-tocopherol were found to be lower than those previously obtained for the present squid waste [28]; differences with the previous study can be explained on the basis that different extraction conditions were employed (i.e., a WW/SV ratio of 0.35 g·mL^−1^) and composition variations resulting from the catching season [44].

Previous research focused on the extraction of tocopherol from marine substrates by low-toxicity solvents. However, the effect of the number of extractions and the substrate weight/solvent volume ratio was not analysed. Thus, tocopherol compound extraction from squid (*D. gahi*) waste with ethanol, acetone, and ethyl acetate was studied by Rodríguez et al. [28]; acetone led to the highest tocopherol (α, γ, and δ compounds) yields while ethanol led to the lowest recovery values. An important effect of low-toxicity solvent polarity was also detected by Gigliotti et al. [24] during Antarctic krill (*E. superba*) extraction; such a study revealed an increasing tocopherol yield by increasing the acetone ratio in an acetone/ethanol extracting system. Recently, Li et al. [25] obtained a higher α-tocopherol recovery from microalga *S. dimorphus* by employing ethanol-containing systems (aq. 95% ethanol; ethanol/hexane, 3:2, *v*/*v*) than in the case of using ethyl acetate/hexane (1:1, *v*/*v*), methanol/hexane (1:0.8, *v*/*v*), or hexane alone.

### 3.5. Effect of the Extracting Conditions on the FA Composition

The FA profile of the lyophilized waste obtained by the conventional extraction procedure revealed the following composition (g·100 g^−1^ total FAs): 3.65 ± 0.04 (C14:0), 0.48 ± 0.03 (C15:0), 22.81 ± 0.26 (C16:0), 1.64 ± 0.02 (C16:1ω7), 0.84 ± 0.03 (C17:0), 3.94 ± 0.07 (C18:0), 3.61 ± 0.11 (C18:1ω9), 2.57 ± 0.09 (C18:1ω7), 0.57 ± 0.05 (C18:2ω6), 4.45 ± 0.04 (C20:1ω9), 0.47 ± 0.11 (C20:2ω6), 2.65 ± 0.03 (C20:4ω6), 0.57 ± 0.03 (C22:1ω9), 17.35 ± 0.01 (C20:5ω3), 0.26 ± 0.05 (C22:4ω6), 0.67 ± 0.04 (C24:1ω9), 0.56 ± 0.06 (C22:5ω3), and 32.90 ± 0.20 (C22:6ω3). If FA groups are considered, the following composition was obtained (g·100 g^−1^ total FAs): 31.72 ± 0.34 (STFAs), 13.51 ± 0.21 (MUFAs), 54.77 ± 0.13 (PUFAs), 50.81 ± 0.14 (total ω3 FAs), and 3.96 ± 0.10 (total ω6 FAs).

Previous research on the waste of the present squid species has also shown DHA, C16:0, and EPA as the most abundant FAs [27,44]. Additionally, the PUFA group was the most abundant in the lipid fraction of the current waste, while MUFA content showed to be low according to previous related research [27,44]. Remarkably, ω3 PUFAs showed to be especially abundant in total PUFAs.

The current FA composition can be considered similar to the one reported in previous research for edible parts of cephalopod species and marine species in general [34,45,46]. Nowadays, great attention is being paid to the presence of EPA, DHA, and ω3 FAs in general, in agreement with their beneficial health effects [7,41,42]. Based on epidemiological and clinical studies, EPA consumption has been related to a low prevalence of circulatory, inflammatory, and coronary diseases [3], while DHA has been associated with the prevention of neurodegenerative diseases, foetal development, and correct functioning of the nervous system and visual organs in the foetus [4].

Therefore, the individual FA analysis of the present study will be focused on the contents of both ω3 FAs. The contents of both ω3 FAs obtained by ethanol/acetone extraction of the present squid waste are shown in Table 3.

For the EPA content, the effect of the WW/SV ratio and the NoE did not lead to significant differences (*p* > 0.05). All EPA values were included in a very narrow range (17.38–17.73 g·100 g^−1^ total FAs), which was very close to the value obtained with the conventional procedure (17.82 g·100 g^−1^ total FAs). In the case of the DHA content, some differences were observed among the different ethanol/acetone conditions tested. Thus, a decreased (*p* < 0.05) DHA presence was detected by increasing the NoE in the case of WW/SV values of 0.25 and 0.30 g·mL^−1^. For the WW/SV ratio, an increased value led to an increased DHA presence (*p* < 0.05) in lipid extracts corresponding to two-time extractions. Similar to EPA content, DHA values for all ethanol/acetone extracting conditions were included in a narrow range (34.09–34.93 g·100 g^−1^ total FAs); such a range was found to be higher than the value obtained for the conventional procedure (33.13 g·100 g^−1^ total FAs).

Previous related research analysed the EPA and DHA presence in lipid extracts obtained from squid (*D. gahi*) waste with different kinds of low-toxicity solvents (ethanol, acetone, ethyl acetate, and binary mixtures) [27]. Thus, the employment of ethanol/acetone (50:50, *v*/*v*) led to values of 15.82 and 31.92 g·100 g^−1^ total FAs for both ω3 FAs, respectively. Such values were similar to those obtained in the conventional extraction procedure (16.05 and 31.12 g·100 g^−1^ FAs) and were found to be lower than those reported in Table 3. Differences with the present study can be explained on the basis that different extracting conditions were employed (i.e., a WW/SV value of 0.35 g·mL^−1^) and that the lipid composition of the squid waste varied with the seasons.

Previous research accounts for EPA and DHA values in lipid extracts of marine waste obtained by different kinds of eco-friendly extracting methods. Such values have been shown to depend on the species and the part of the body taken into account. Compared to current results, lower levels of DHA (25.5 g·100 g^−1^ total FAs) were obtained by Chantachum et al. [36] during a comparative study on crude oil extraction in precooked and non-precooked skipjack tuna (*K. pelamis*) heads by a wet-reduction method; in the meantime, negligible levels of EPA were obtained in both kinds of samples. Lower EPA (7.1–7.4 g·100 g^−1^ total FAs) and DHA (8.0–8.3 g·100 g^−1^ total FAs) contents than in the present study were detected in three different salmon (*S. salar*) by-products (skins, heads, and backbones) when extracted following different eco-friendly methods (95 °C for 30 min, <15 °C, and enzyme-assisted extraction) followed by pressing and centrifugation [43]. A high EPA (5–6%), DHA (31–33%), and total PUFA (40–41 g·100 g^−1^ total FAs) yield was obtained from skipjack (*K. pelamis*) eyeballs by employing a wet-rendering method [20]; as in the present case, MUFAs showed the lowest content among FA groups (ca. 21%). Increased EPA, DHA, and PUFA levels were obtained by ethanol/hexane (2:1, *v*/*v*) extraction on hoki roe by comparison to the conventional Folch method [37]; on the contrary, lower MUFA levels were obtained than in the conventional procedure. Recently, lower EPA (9.3 g·100 g^−1^ total FAs) and DHA (16.4 g·100 g^−1^ total FAs) contents than in the present study were obtained by Rodríguez et al. [38] by employing different conditions of wet-pressing extraction on Argentinean shortfin squid (*I. argentinus*) viscera.

### 3.6. Effect of the Extraction Conditions on the FA Ratios

For all kinds of marine substrates, great attention has been accorded to certain FA ratios on the basis of their direct relationship with human health enhancement. This attention concerns the PUFA/STFA and ω3/ω6 ratios for their direct relationship with nutritional and digestibility values and with health preservative properties [2,4,47]. In an attempt to avoid health concerns such as cardiovascular, neurological, and inflammatory disorders, the World Health Organisation (WHO) recommends that the ω3/ω6 ratio should not be below 1:10 in the human diet [48]. Similarly, the European Nutritional Society reported that a human diet with an ω3/ω6 ratio of 1:5 or higher would lead to health benefits [1]. Therefore, values obtained in the present study for the PUFA/STFA and ω3/ω6 ratios will be analysed and discussed in the following lines.

**Figure 2:**
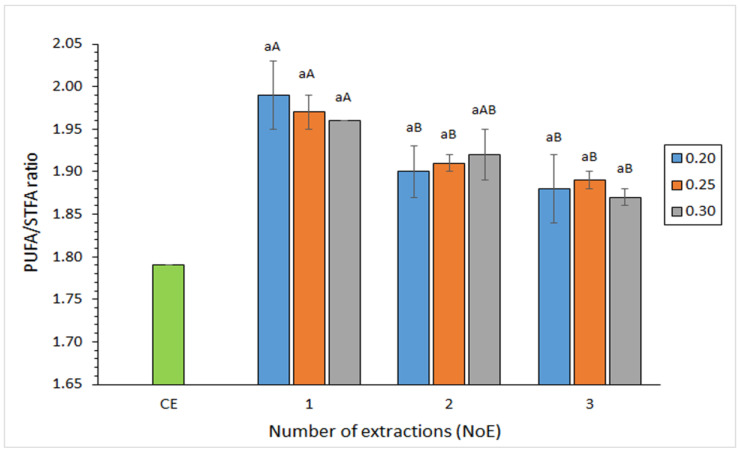
Effect of ethanol/acetone extraction conditions on the PUFA/STFA ratio in the lipid fraction obtained from squid waste. Average values of four replicates (*n* = 4); standard deviations are indicated by bars. For each WW/SV ratio (0.20, 0.25, or 0.30), different capital letters (A,B) denote significant differences (*p* < 0.05) as a result of the NoE. No effect (*p* > 0.05) of the WW/SV ratio (0.20, 0.25, or 0.30 g·mL^−1^) was detected (lowercase letter “a” in all cases). Abbreviations: CE (conventional extraction), WW/SV (waste weight/solvent volume; g·mL^−1^), NoE (number of extractions), PUFA (polyunsaturated fatty acids), and STFA (saturated fatty acids).

In all present extracts obtained by the ethanol/acetone solvent mixture, PUFA/STFA average values were included in a narrow range (1.87–1.99) and were found to be higher than the one detected in the lipid extract corresponding to the conventional procedure (1.79) (Figure 2). An increased NoE led to a progressive decrease (*p* < 0.05) of the PUFA/STFA ratio. This result is in agreement with the above-mentioned tendency obtained for the DHA presence in the ethanol/acetone extracts. No effect (*p* > 0.05) of the WW/SV value could be concluded on the PUFA/STFA ratio.

As for the PUFA/STFA ratio, all ω3/ω6 average values corresponding to the ethanol/acetone extraction system were included in a narrow range (12.24–13.00). Average values were found to be higher in most cases than those detected in the lipid extract obtained by the conventional procedure (Table 3). A decreasing ω3/ω6 value (*p* < 0.05) was obtained by increasing the NoE in samples, corresponding to a WW/SV value of 0.25. Additionally, a higher (*p* < 0.05) ω3/ω6 value was detected in samples, corresponding to a one-time extraction when considering a WW/SV value of 0.25 g·mL^−1^.

The present ω3/ω6 values are in agreement with those obtained in the present squid waste by employing different low-toxicity solvent systems (ethanol, acetone, ethyl acetate, and binary mixtures) [27]. In such a study, ω3/ω6 values included in the 11–14 range were obtained, with ethanol/acetone extraction (WW/SV ratio of 0.35 g·mL^−1^ and a three-time extraction) leading to an average value of ca. 13.

Previous studies on waste substrates corresponding to the processing of other marine species have also focused on the ω3/ω6 ratio. Thus, Šimat et al. [49] obtained values included in the 6–10 range for tuna (*Thunnus thynnus*) and sardine (*Sardina pilchardus*) by-products, while markedly lower ratios (0–2 range) were obtained for tuna (*T. thynnus*) liver and sea bass (*Sparus aurata*) and sea bream (*Dicentrarchus labrax*) by-products. An ω3/ω6 value of 12.5 was obtained by Ahmmed et al. [37] by employing an ethanol/hexane (2:1, *v*/*v*) extraction on hoki roe; this value was found to be similar to the one obtained in the lipid extracts resulting from the conventional Folch method and the two-step ethanol/hexane procedure. The ω3/ω6 ratio of lipid extracts obtained by different wet-pressing procedures on squid (*I. argentinus*) viscera was analysed by Rodríguez et al. [38]; as a result, lower ω3/ω6 values (7.0–8.0 range) than in the present study were detected and were also lower than those levels obtained by the conventional extraction procedure (i.e., 8.6).

## 4. Conclusions

An increased NoE led to an increased (*p* < 0.05) TL yield but a decreased (*p* < 0.05) proportion of PLs in the lipid extract. Additionally, a lower (*p* < 0.05) PUFA/STFA ratio was detected by increasing the NoE. Some differences (*p* < 0.05) could be outlined as a result of increasing or decreasing the WW/SV ratio; however, a definite trend for this extraction condition could not be concluded for any of the valuable lipid compounds taken into account. Compared to the conventional chloroform/methanol procedure, a 94–99% TL recovery was accomplished provided a three-time extraction was carried out. Meantime, a high PL recovery (98–106%) was observed when compared to the lipid extract obtained by the conventional procedure; notably, higher average values (over 100%) were obtained if a one-time extraction was carried out. No effect (*p* > 0.05) of the NoE was observed on the α- and γ-tocopherol yields; remarkably, all ethanol/acetone extracts led to recovery yields similar to those obtained by the conventional procedure.

It is concluded that, in order to attain an increased yield, the NoE required would depend on the polarity degree of the compound concerned. Thus, an increased extraction of TLs would require a three-time extraction. Contrary to popular belief, to obtain a highly PL-concentrated extract, a one-time extraction ought to be employed. To obtain a lipid extract showing a high PUFA/STFA ratio, a one-time extraction ought to be carried out.

The effect of two extracting conditions (NoE and WW/SV ratio) was analysed, to our knowledge for the first time, during the lipid extraction of a marine substrate by employing a low-toxicity solvent mixture. Present results confirm the suitability of the ethanol/acetone (50:50, *v*/*v*) mixture as a low-toxicity solvent procedure to be employed as an alternative method for the extraction of lipid compounds. Remarkably, all ethanol/acetone extracting conditions tested led to valuable yields for lipid molecules (PLs, α-tocopherol, and ω3 FAs) and FA ratios (PUFAs/STFAs and ω3/ω6) with healthy, nutritional, and preserving properties. Further research is envisaged to check the suitability of the present low-toxicity solvent mixture for the extraction of valuable lipids from other kinds of marine waste substrates.

## Figures and Tables

**Table 1:** Extraction conditions (from EC-1 to EC-9) employed for the lipid extraction of squid waste with an ethanol/acetone (50:50, *v*/*v*) mixture.

Extraction Condition Number	Experimental Conditions		Process Variables	
	Waste Weight (WW) (g)	Solvent Volume (SV) (mL)	WW/SV Ratio (g·mL^−1^)	Numer of Extractions (NoE)
EC-1	3	15	0.20	1
EC-2	3	12	0.25	1
EC-3	3	10	0.30	1
EC-4	3	15	0.20	2
EC-5	3	12	0.25	2
EC-6	3	10	0.30	2
EC-7	3	15	0.20	3
EC-8	3	12	0.25	3
EC-9	3	10	0.30	3

**Table 2:** Effect of ethanol/acetone extraction conditions * on phospholipid (PL; g·kg^−1^ lipids) and α- and γ-tocopherol (mg·kg^−1^ lipids) yields in the lipid fraction obtained from squid waste **.

Extraction Condition		Lipid Parameter		
WW/SV (g·mL^−1^)	NoE	PLs	α-Tocopherol	γ-Tocopherol
0.20	1	343.0 aA (6.6)	624.8 bA (44.6)	10.4 abA (1.5)
0.25	1	343.9 aA (7.0)	699.0 aA (11.8)	15.4 aA (3.0)
0.30	1	334.6 aA (4.8)	643.4 abA (23.9)	9.4 bAB (0.5)
0.20	2	329.2 bA (9.0)	687.5 aA (23.5)	10.9 aA (0.2)
0.25	2	322.7 bB (2.2)	593.4 abB (8.5)	7.8 bB (0.3)
0.30	2	341.3 aA (13.4)	575.7 bB (24.0)	7.8 bB (0.2)
0.20	3	328.5 aA (4.7)	667.6 aA (19.7)	10.3 aA (1.3)
0.25	3	315.5 bB (6.9)	596.1 aB (70.1)	8.1 aB (0.7)
0.30	3	316.3 abA (19.8)	647.9 aA (21.1)	8.9 aA (0.1)
Conventional extraction		323.2 (3.2)	617.4 (82.4)	8.1 (2.4)

* Average values of four replicates (*n* = 4); standard deviations are indicated in brackets. For each NoE, different lowercase letters (a,b) denote significant (*p* < 0.05) differences as a result of the WW/SV ratio. For each WW/SV ratio, different capital letters (A,B) denote significant differences (*p* < 0.05) as a result of the NoE. ** Abbreviations: WW/SV (waste weight/solvent volume) and NoE (number of extractions).

**Table 3:** Effect of ethanol/acetone extraction conditions * on EPA and DHA content (g·100 g^−1^ total FAs) and ω3/ω6 ratio in the lipid fraction obtained from squid waste **.

Extraction Condition		FA Parameter		
WW/SV (g·mL^−1^)	NoE	EPA	DHA	ω3/ω6
0.20	1	17.73 aA (0.39)	34.67 aA (0.33)	12.24 bA (0.36)
0.25	1	17.52 aA (0.08)	34.93 aA (0.14)	12.91 aA (0.00)
0.30	1	17.40 aA (0.20)	34.76 aA (0.08)	12.38 bA (0.39)
0.20	2	17.50 aA (0.04)	34.29 bA (0.07)	12.90 aA (0.74)
0.25	2	17.48 aA (0.21)	34.52 abB (0.14)	12.62 aB (0.02)
0.30	2	17.36 aA (0.08)	34.56 aA (0.14)	12.67 aA (0.53)
0.20	3	17.38 aA (0.17)	34.09 aA (0.18)	12.64 aA (0.34)
0.25	3	17.39 aA (0.11)	34.25 aC (0.07)	12.26 aC (0.25)
0.30	3	17.38 aA (0.21)	34.33 aB (0.02)	13.00 aA (0.52)
Conventional extraction		17.82 (0.00)	33.13 (0.00)	12.38 (0.00)

* Average values of four replicates (*n* = 4); standard deviations are indicated in brackets. For each NoE, different lowercase letters (a,b) denote significant (*p* < 0.05) differences as a result of the WW/SV ratio employed. For each WW/SV ratio, different capital letters (A,B,C) denote significant differences (*p* < 0.05) as a result of the NoE. ** Abbreviations: WW/SV (waste weight/solvent volume), NoE (number of extractions), FA (fatty acid), EPA (eicosapentaenoic acid), and DHA (docosahexaenoic acid).

## Data Availability

Not applicable.
